# Selective Genicular Artery Embolisation for Recurrent Hemarthrosis Following Total Knee Arthroplasty: A Case Report

**DOI:** 10.7759/cureus.98179

**Published:** 2025-11-30

**Authors:** Aakanksha Garlapati, Shahmeen Rasul, Neil Ashwood, Keith Hayward

**Affiliations:** 1 Trauma and Orthopaedics, University of Leicester, Newcastle-under-Lyme, GBR; 2 Trauma and Orthopaedics, University Hospitals of Derby and Burton National Health Service (NHS) Foundation Trust, Derby, GBR; 3 Trauma and Orthopaedics, University Hospitals of Derby and Burton National Health Service (NHS) Foundation Trust, Burton, GBR

**Keywords:** anticoagulation, genicular artery embolisation, recurrent hemathrosis, synovial hypervascularity, total knee replacement (tkr)

## Abstract

Recurrent hemarthrosis following total knee arthroplasty (TKA) is an uncommon complication, with an incidence of less than 1%. It can lead to pain, swelling, joint stiffness, and functional impairment. Selective genicular artery embolisation (GAE) has emerged as a minimally invasive treatment for managing such cases. We report the case of a 79-year-old female with recurrent atraumatic hemarthrosis of the left knee following TKA, complicated by long-term anticoagulation with Edoxaban for unprovoked pulmonary embolism. Despite multiple aspirations and arthroscopic washouts, symptoms persisted. Angiography revealed synovial hypervascularity, and GAE was performed to reduce bleeding. Although the procedure initially improved symptoms, recurrence occurred while the patient was maintained on full-dose Edoxaban. Following a multidisciplinary review, the anticoagulant dose was reduced, resulting in complete resolution of haemarthrosis. GAE represents an effective, minimally invasive treatment for recurrent hemarthrosis post-TKA. Anticoagulation management and multidisciplinary coordination are essential to balance bleeding risk and thromboembolic protection in such patients.

## Introduction

Genicular artery embolization (GAE) is a minimally invasive process that is designed to reduce the pain and inflammation, which is caused by hemarthrosis of the knee joint. GAE is used for recurrent hemarthrosis following total knee arthroplasty (TKA); however, it is a newly emerging treatment for pain due to osteoarthritis if the patient is not fit for surgery [1].

The genicular arteries are six arteries in the human leg, five of which are branches of the popliteal artery and one that branches from the femoral artery. The genicular arteries anastomose in the knee region and supply blood not only to the articular joint but also to the surrounding skin, ligaments, tendons, muscles, and bone structures. GAE involves selectively catheterising the genicular arteries that supply the knee’s synovial lining during an angiogram. The procedure targets the hypervascularity of the synovial arteries within the knee joint, aiming to decrease inflammation-related knee pain [2-4].

Hemarthrosis is a condition that includes articular bleeding (into the joint cavity). Recurrent hemarthrosis is a rare complication after TKA, with an incidence reported as less than 1%. It can present with persistent pain, stiffness, swelling, and bruising of the knee joint without any associated trauma. The possible causes of recurrent hemarthrosis include anticoagulant therapy, bleeding disorders, pseudoaneurysms, ruptured peripheral artery aneurysms, and pigmented villonodular synovitis [5,6].

GAE following TKA is an uncommon procedure, primarily used to manage rare complications such as recurrent hemarthrosis, synovial hypervascularity, or vascular abnormalities like geniculate artery pseudoaneurysms. Recurrent hemarthrosis occurs in only about 0.3-1.6% of TKA cases, and only a small fraction of those patients ultimately require GAE, making its overall use extremely rare. Current evidence, derived mostly from case reports and small series, suggests that GAE can be an effective, minimally invasive option for select patients with persistent bleeding or vascular complications unresponsive to conventional treatment, such as immobilisation of the joint, and is considered preferable due to its minimally invasive nature and faster recovery time [7,8].

## Case presentation

**Figure 1:**
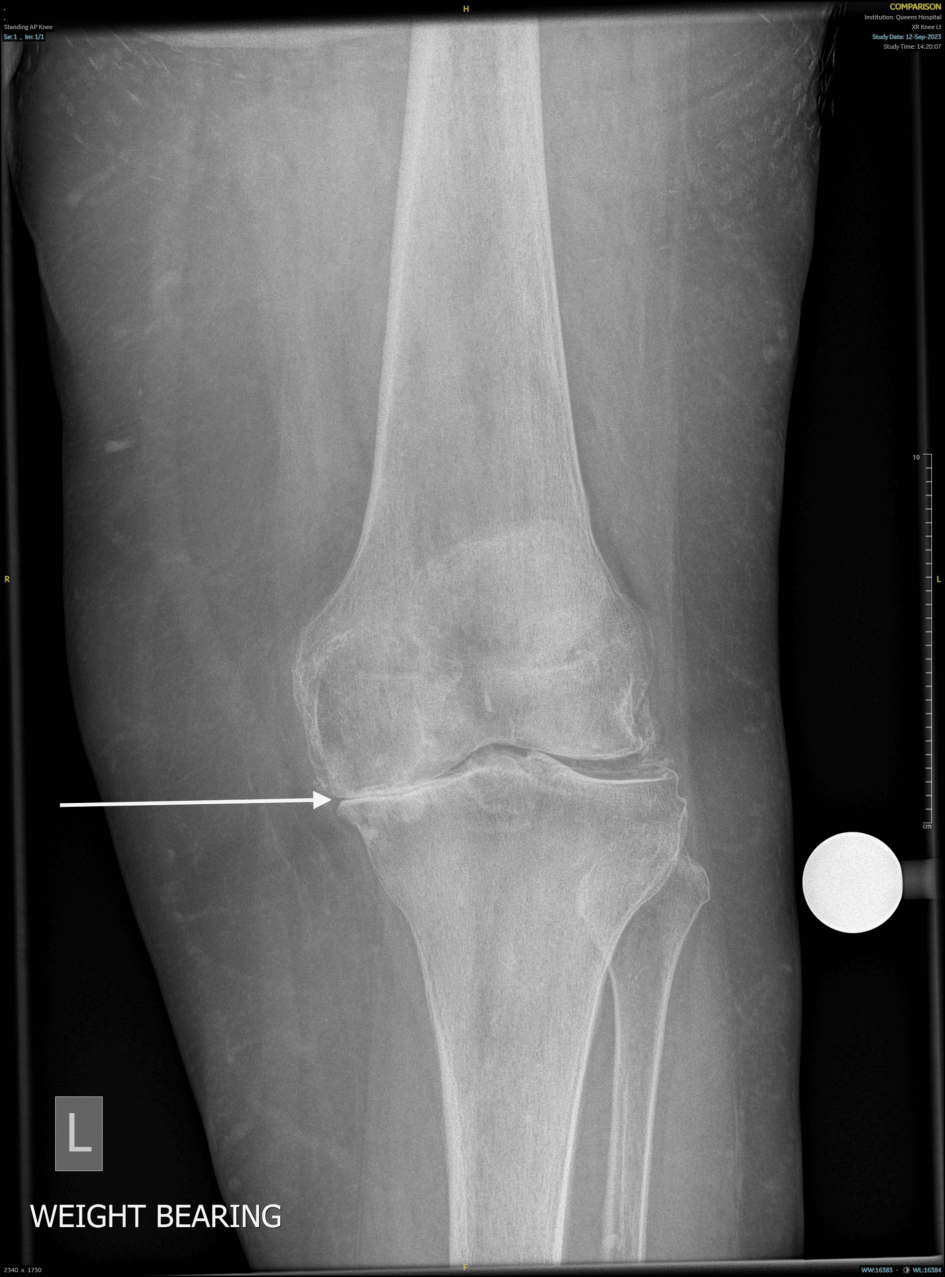
Preoperative anteroposterior radiograph of the left knee showing degenerative changes of the joint prior to total knee replacement The arrow indicates degenerative changes of the knee joint consistent with osteoarthritis.

**Figure 2:**
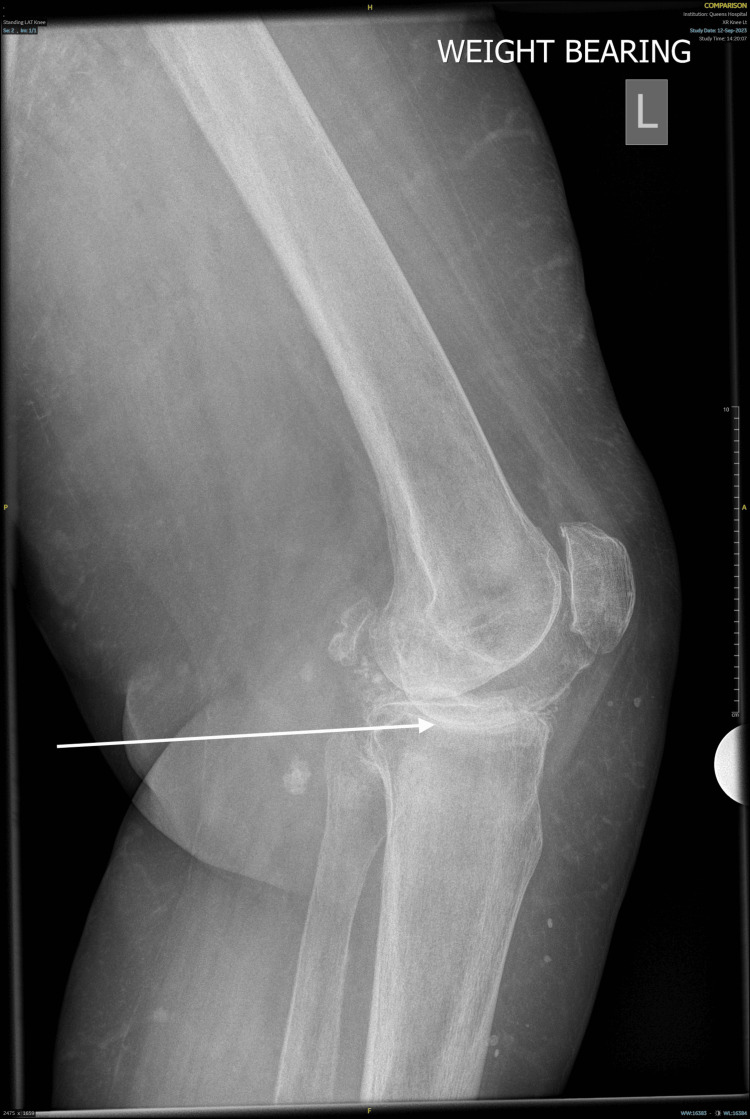
Preoperative lateral radiograph of the left knee showing degenerative changes of the joint prior to total knee replacement The arrow indicates degenerative changes of the knee joint consistent with osteoarthritis.

A 79-year-old woman presented with clinical suspicion of recurrent hemarthrosis following a left total knee replacement (TKR) performed in January 2024. Since the procedure, she has experienced several episodes of acute, painful swelling of the left knee, each associated with ecchymosis, restricted range of motion, and temporary loss of joint function. The left TKR had been indicated for advanced osteoarthritis with significant degenerative changes (Figures 1, 2). Of note, she previously underwent an uncomplicated right TKR several years earlier, which continues to function well without issues.

Her past medical history is notable for unprovoked pulmonary embolism, atrial fibrillation, and hypertension. She is currently anticoagulated with edoxaban for pulmonary embolism management, having been transitioned from warfarin following previous bleeding episodes related to hemarthrosis.

Over the preceding six months, the patient experienced two episodes of sudden, painful swelling of the left knee. She came back in May 2025 for her first orthopaedic intervention, which involved aspiration and arthroscopic washout of the left TKR. Intraoperative findings revealed dark blood upon aspiration, while Synovasure testing was negative, excluding periprosthetic joint infection. Some scar tissue formation was noted; however, the prosthesis appeared well-fixed and stable. The joint was irrigated with 3 liters of saline, resulting in an improvement in knee flexion from 80° to 95°. Postoperatively, the patient was recommended to be fully weight-bearing, and anticoagulation with edoxaban was resumed after 72 hours, with bridging enoxaparin administered in the interim.

The patient re-presented with pain and swelling of the left leg two months later. Given her recent knee surgery and history of unprovoked pulmonary embolism, a deep vein thrombosis (DVT) was suspected. Duplex ultrasonography demonstrated no evidence of DVT; however, a haemorrhagic Baker’s cyst measuring 33 x 15 x 19 mm was identified. Notably, a prior ultrasound performed in January 2025 had already documented the presence of a Baker’s cyst in the same region.

**Figure 3:**
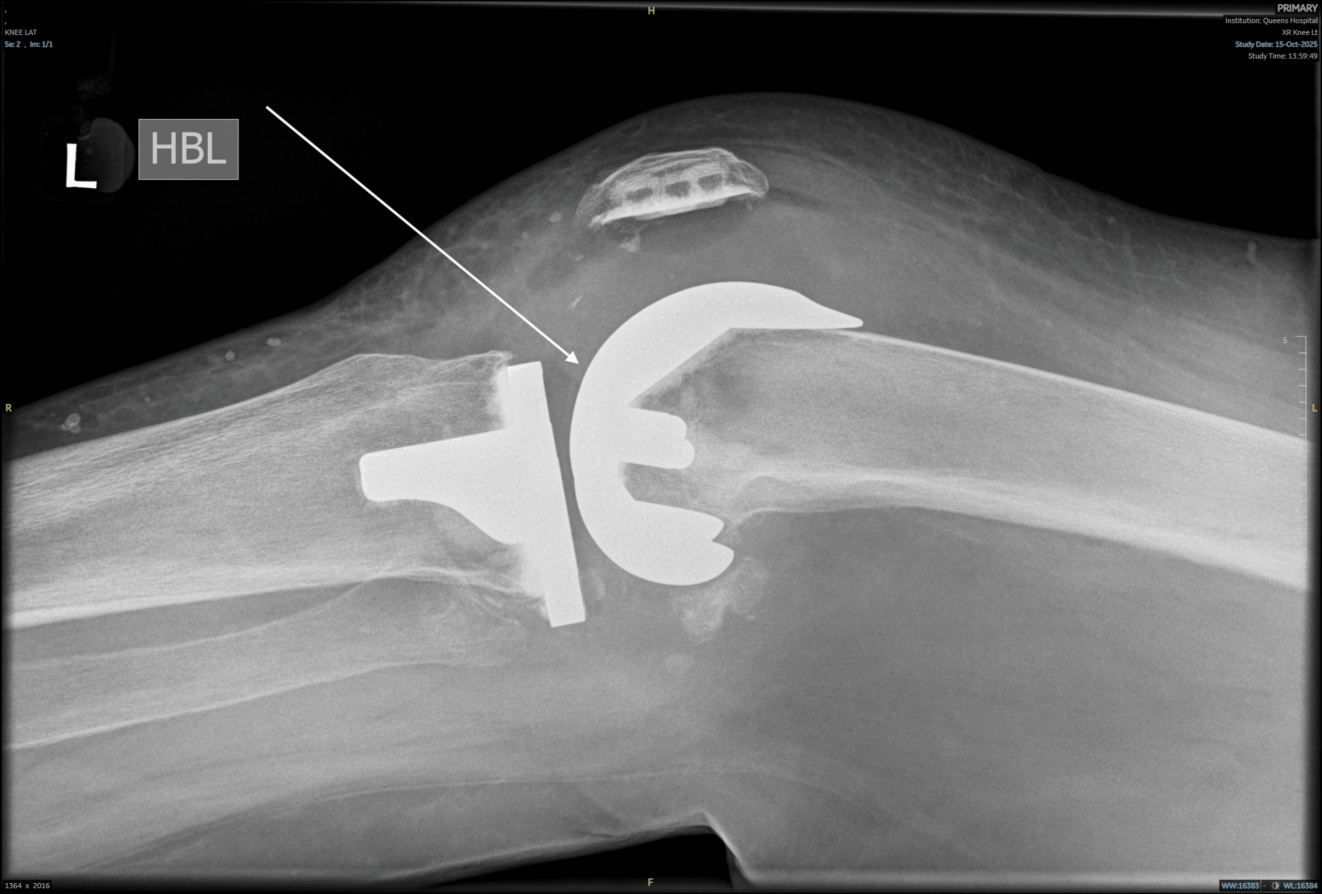
Lateral radiograph of the left knee obtained prior to aspiration, demonstrating the total knee replacement prosthesis in situ. The arrow indicates the total knee replacement prosthesis in situ

The patient subsequently underwent a repeat aspiration of the left knee four months after the first one. An X-ray was taken before the aspiration procedure, which demonstrated the total knee replacement prosthesis (Figure 3). During the procedure, 6 mL of blood was aspirated and sent for microbiological analysis in blood culture bottles. Following aspiration, 1 g of intra-articular tranexamic acid was administered. Haematology review raised concern regarding recurrent atraumatic hemarthrosis in the context of ongoing anticoagulation with edoxaban. Laboratory investigations demonstrated a mildly prolonged prothrombin time of 17.9 seconds (reference range: nine to 13 seconds) and an INR of 1.5 (reference range: 0.8-1.2), findings that may reflect the effect of the direct oral anticoagulant (DOAC). A prior connective tissue disease screen was negative. Renal function revealed mild impairment with an estimated glomerular filtration rate (eGFR) of 74 mL/min (reference value: 90 mL/min), and further evaluation, including liver function tests, was planned to exclude hepatic dysfunction. There was no evidence of systemic bleeding, bruising, or involvement of other joints. In the absence of a clear underlying cause on laboratory testing or imaging, such as CT angiography, a discussion was recommended to evaluate the risks and benefits of continued anticoagulation, balancing the patient’s history of large bilateral unprovoked pulmonary emboli against the potential for recurrent intra-articular bleeding associated with DOAC therapy.

To investigate the cause of bleeding, a CT angiogram of both lower limbs was arranged. Unfortunately, the scan was acquired suboptimally with poor visualisation of the vessels around the knee due to artefact from the metalwork. In the left leg, the common femoral, profunda, and superficial femoral arteries appear normal, but the popliteal artery is largely obscured by severe beam hardening artefact from the knee prosthesis. No obvious pseudo-aneurysm or other arterial pathology, although the genicular arteries cannot be assessed on this study as a result. Allowing for poor contrast opacification, there appears to be reasonable three-vessel run-off in the calf. Similarly, in the right leg, the arterial tree appears normal, although the popliteal artery is largely obscured by beam hardening artefact from the knee prosthesis. There is distension of the long saphenous veins bilaterally, with varicose veins noted, particularly in the calves.

**Figure 4:**
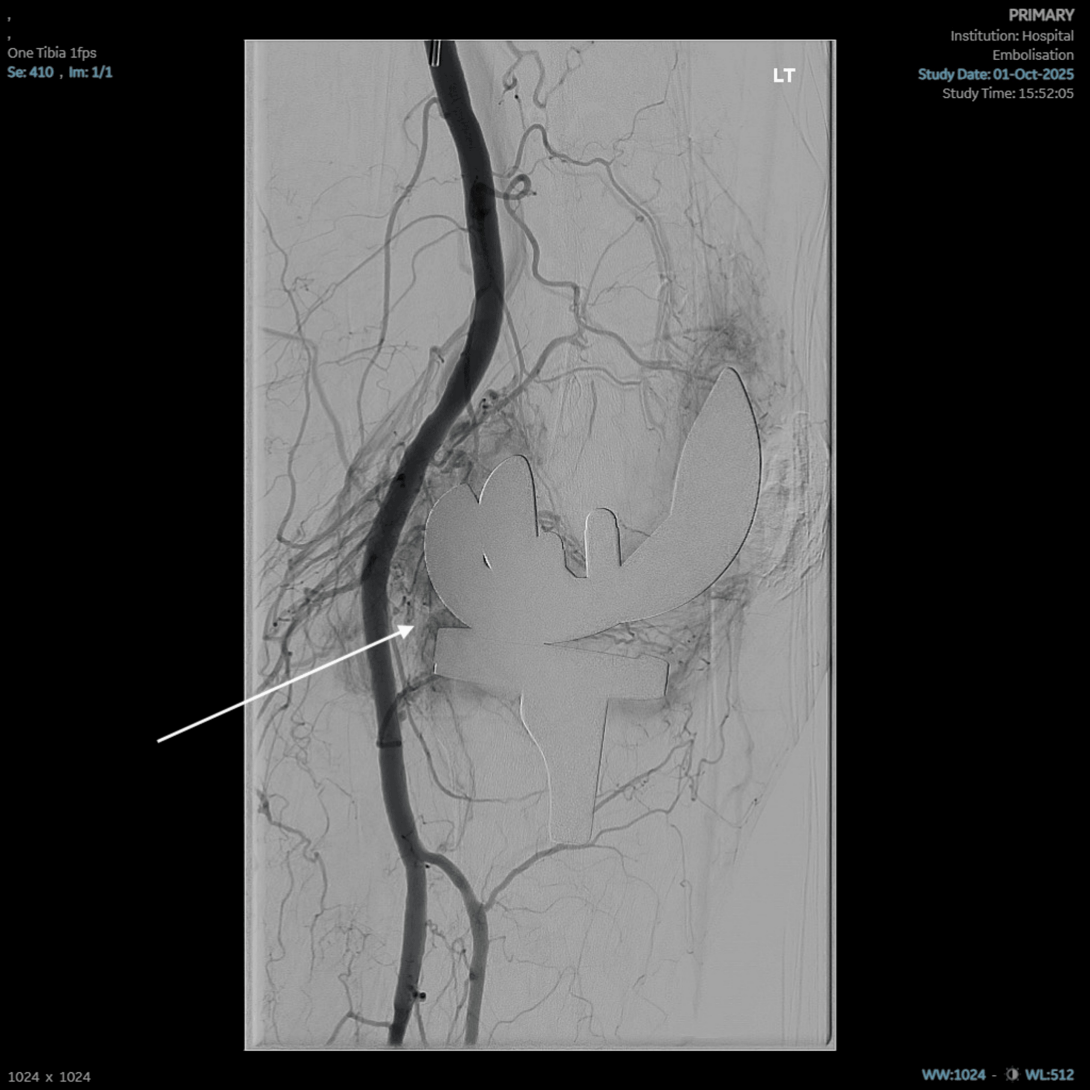
Angiography demonstrates areas of synovial hypervascularity around the knee joint without evidence of pseudoaneurysm or vessel injury. The arrow highlights areas of synovial hypervascularity demonstrated on angiography.

**Figure 5:**
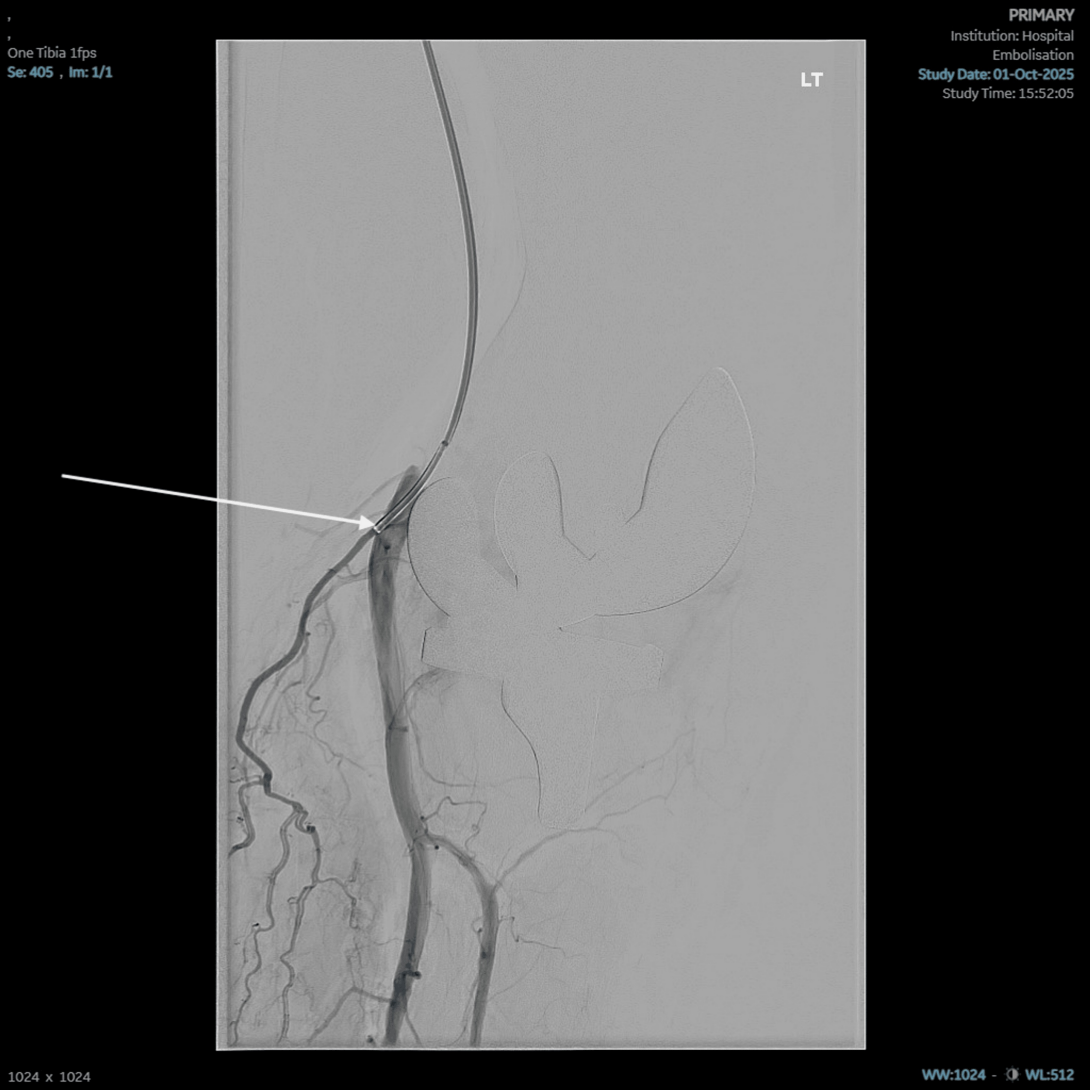
The Direxion microcatheter is visualised within the target vessel. The arrow highlights the Direxion microcatheter within the target vessel.

Following discussions between the consultant orthopaedic surgeon and consultant radiologist, the plan was for fluoroscopic-guided embolisation of genicular vessels in the left knee. The procedure took place in early October 2025. Angiography demonstrates areas of synovial hypervascularity around the knee joint but no evidence of pseudoaneurysm or vessel injury (Figure 4). Selective angiography was performed of multiple genicular vessels, which were catheterised using a Direxion microcatheter (manufactured by Boston Scientific, Massachusetts, United States). A vessel leading to significant synovial enhancement was embolised using Embosphere Microsphere 400 µm particles (supplied by Merit Medical Systems, Utah, United States), and a further tortuous descending genicular artery was occluded using a microcatheter. Completion angiography demonstrates improved appearances with less visible hypervascularity in relation to the knee joint (Figure 5). There were no immediate complications.

Two weeks after embolisation, the patient re-presented with recurrent pain and swelling of the left knee in the absence of any reported trauma. Clinical examination revealed moderate effusion with a reduced range of motion limited to 40°, while distal pulses and sensation remained intact. Laboratory investigations demonstrated a mildly elevated D-dimer level of 373 µg/L (reference range: <250 µg/L), without evidence of infection or acute DVT. The management plan was conservative, with temporary cessation of Edoxaban in case of potential joint washout and initiation of low-molecular-weight heparin (LMWH) for venous thromboembolism (VTE) prophylaxis.

The patient was managed conservatively without further surgical intervention or joint aspiration. She remained an inpatient for five days, during which a discussion with the haematology team was held. It was agreed that lifelong VTE prophylaxis was indicated, given her history of unprovoked bilateral pulmonary emboli. Haematology recommended a trial of reduced-dose DOAC therapy to mitigate the risk of recurrent hemarthrosis. Consequently, her edoxaban dose was halved, after which no further episodes of hemarthrosis occurred during the admission. The patient was subsequently deemed medically stable and discharged home in an optimised condition.

## Discussion

The incidence of recurrent hemarthrosis after TKR ranges from 0.1% to 1.6% [9]. The sequelae of recurrent hemarthrosis in knee replacement can include pain, joint stiffness, reduced range of motion, and an increased risk of infection [10]. Reported causes include anticoagulant therapy, bleeding disorders, pseudoaneurysm formation, ruptured peripheral artery aneurysms, and pigmented villonodular synovitis [6].

Several studies have explored the use of GAE to manage bleeding secondary to synovial hypertrophy in cases of recurrent hemarthrosis [11]. In one study, six of seven patients achieved complete symptom resolution following embolisation [12]. Another series of 13 patients undergoing 14 GAE procedures for recurrent hemarthrosis after TKR reported clinical success in 85.7% of cases, with an average of 3.6 genicular vessels embolised per patient [13].

In the present case, recurrent atraumatic hemarthrosis of the left TKR was attributed to synovial hypervascularity, exacerbated by long-term anticoagulation with edoxaban. The CT angiogram was non-diagnostic due to the metal artefact, highlighting the importance of obtaining diagnostically optimal angiography when evaluating vascular causes of prosthetic joint hemarthrosis. Embolisation effectively reduced synovial vascularity; however, recurrence occurred while the patient remained on full-dose therapy. Symptom resolution following dose reduction highlights the delicate balance between maintaining thromboembolic protection and minimising bleeding risk in anticoagulated patients with prosthetic joints.

## Conclusions

Recurrent hemarthrosis following TKA is a rare but significant complication that may be exacerbated by anticoagulation therapy. Selective genicular artery embolisation offers a minimally invasive treatment option to control the synovial hypervascularity and reduce the bleeding. However, recurrence is a risk if the patient requires ongoing anticoagulation. This case emphasises the importance of individual management and highlights the value of multidisciplinary collaboration to optimise patient outcomes.
